# Effect of the *CCND1* A870G polymorphism on prostate cancer risk: a meta-analysis of 3,820 cases and 3,825 controls

**DOI:** 10.1186/s12957-015-0479-8

**Published:** 2015-02-15

**Authors:** Min Zheng, Lijun Wan, Xiang He, Xiaolong Qi, Feng Liu, Da-Hong Zhang

**Affiliations:** Department of Urology, Zhejiang Provincial People’s Hospital, No.158 Shangtang Road, Hangzhou, Zhejiang 310014 China; Department of Urology, Quzhou People’s Hospital, No. 2 Zhongloudi Street, Quzhou, 310014 China

**Keywords:** *CCND1* A870G, Polymorphism, Prostate cancer

## Abstract

**Background:**

*Cyclin D1* (*CCND1*) is critical in the transition of the cell cycle from the G1 to S phases, and unbalanced cell cycle regulation is a hallmark of carcinogenesis. Numerous epidemiological studies have evaluated the association between the *CCND1* A870G polymorphism and the risk of prostate cancer (PCa). However, these studies have yielded conflicting results.

**Methods:**

In the present study, the possible association above was assessed by a meta-analysis. Eligible articles were identified for the period up to July 2014. Pooled odds ratios (ORs) with 95% confidence intervals (95% CI) were appropriately derived from fixed effects or random effects models.

**Results:**

A total of ten case-control studies, which included 3,820 cases and 3,825 controls, were identified. Overall, the allelic/genotypic association between the G870A polymorphism and prostate cancer was nonsignificant (OR = 1.045, 95% CI = 0.947 to 1.153 for A versus G, *P* = 0.380; OR = 1.088, 95% CI = 0.896 to 1.321 for AA versus GG, *P* = 0.393; OR = 1.044, 95% CI = 0.941 to 1.158 for GA versus GG, *P* = 0.414; OR = 1.053, 95% CI = 0.955 to 1.161 for the dominant model AA + GA versus GG, *P* = 0.303; OR = 1.072, 95% CI = 0.881 to 1.306 for the recessive model AA versus AA + GA, *P* = 0.486). Moreover, subgroup analyses according to ethnicity failed to demonstrate a significant association between this polymorphism and prostate cancer. In addition, we also performed a stratified analysis of cases with PCa metastasis, and the results supported the findings of no significant association between *CCND1* A870G polymorphism and metastasis risk of PCa.

**Conclusions:**

Our results suggest that the CCND1 A870G polymorphism might not be a potential candidate for predicting prostate cancer risk, including metastasis risk.

## Background

As one of the most frequent malignant diseases among men, prostate cancer (PCa) is a complex disease that is caused by a multitude of factors [1]. Recently, studies have revealed that genetic factors play an important role in the development of sporadic prostate cancer [2,3], which might provide a potential method for targeted therapy of PCa. Therefore, there is increasing interest in the role that genetic variants such as single nucleotide polymorphic variants (SNPs) play in prostate cancer risk.

Cell cycle dysregulation plays a critical role in a variety of malignancies and contributes to an increased risk of metastasis, in part, by altering the cell’s ability to respond appropriately to DNA damage [4,5]. A number of studies have linked alleles in these genes to increased cancer risk or decreased survival in prostate cancer [6-10]. Cyclin D1, also known as CCND1, is localized to the nucleus and plays a key role in the transition from the G1 to S phase, which promotes the progression of the cell cycle during cell division [11]. The overexpression of CCND1 has always been observed in numerous types of malignant cancer and indicates a poor clinical outcome [12-14]. *Cyclin D1* mRNA is alternatively spliced to transcribe two different transcripts, which yield to functionally different proteins [15,16], and an A870G polymorphism in exon4 of *CCND1* is involved, alternatively, in splicing of *CCND* mRNA [15].

Epidemiological studies have reported the association between the *CCND1* A/A genotype and the risk of various cancers. Qin *et al*. found that the *CCND1* A/A genotype may increase brain tumor risk, especially for gliomas [17]. Yang *et al.* also reported that an A870G polymorphism in *CCND1* confers an increased risk for breast cancer [18]. However, He *et al.* revealed that a *CCND1* G870A polymorphism has no association with esophageal cancer risk in ethnicity and histology [19]. Although a number of studies have been performed to assess the association between the *CCND1* A870G polymorphism and prostate cancer susceptibility, the conclusions have been inconsistent. Wang *et al.* reported that the A allele of the *CCND1* A870G polymorphism was recessively associated with susceptibility to PCa [10]. But no association between the *CCND1* A870G polymorphism and prostate cancer was found by Chen *et al.* [20].

In the present study, ten case-control studies on the *CCND1* A870G polymorphism and prostate cancer risk, which were previously published, were analyzed via a meta-analysis to examine a more specific association between the *CCND1* A870G polymorphism and prostate cancer risk and various published observational studies.

## Methods

### Literature search

The PubMed, MEDLIN, and Web of Science databases were searched for studies published before July 2014. The key words used for searching were as follows: *CCND1*/*cyclin D1*, prostate cancer/carcinoma/tumor, variant/genotype/polymorphism/SNP, and the combined phrases for all genetic studies on the association between the *CCND1* A870G polymorphism and prostate cancer risk. We also checked the references of the retrieved studies and reviews to ensure the complement of this meta-analysis. This work was approved by the ethics committees of Zhejiang Provincial People’s Hospital.

### Inclusion criteria

The following inclusion criteria were used for the literature selection in our meta-analysis: (a) case-control study, (b) evaluation of the *CCND1* polymorphism and prostate cancer risk, and (c) sufficient published data for both patients and controls.

### Exclusion criteria

The following exclusion criteria were set: (1) incomplete raw data, (2) repetitive reports (if studies had partly or completely overlapping data, only the largest or most recent sample was selected), (3) materials and methods were not well-described and reliable, or (4) not an English paper.

### Data collection

Two investigators extracted information independently according to the inclusion and exclusion criteria listed. When it came to conflicting evaluations, an agreement was reached after a discussion. For each eligible study, we collected information as follows: the first author’s name, publication data, country of origin, sources of controls, number of different genotypes, and ethnicity of the study population (categorized as Asian and Caucasian). For studies including subjects of different ethnic groups, data were extracted separately for each ethnic group whenever possible.

### Statistical analysis

The Hardy-Weinberg equilibrium (HWE) for the control group of each study was assessed using a goodness-of-fit test (*χ*^2^ of Fisher’s exact test). Heterogeneity and cumulative analysis were assessed by *χ*^2^-based *Q* test. OR estimation was calculated with the fixed effect model (Mantel-Haenszel method) when statistical heterogeneity did not exist (*P* > 0.10). Otherwise, the random effects model (DerSimonian and Laird method) was selected. Crude odds ratios (ORs) with 95% confidence intervals (CIs) were used to assess the strength of association between the *CCND1* polymorphism and prostate cancer risk. The pooled ORs were performed for a codominant model (GA versus GG and AA versus GG), dominant model (GA/AA versus GG), and recessive model (AA versus GA/GG). Stratified analyses were also performed by ethnicity. Publication bias was evaluated by Funnel plots, and Begg’s test and Egger’s test were also used to detect publication bias. Statistical analysis was performed using STATA versions 11.0 (StataCorp, College Station, TX, USA).

## Results

### Study characteristics

**Figure 1:**
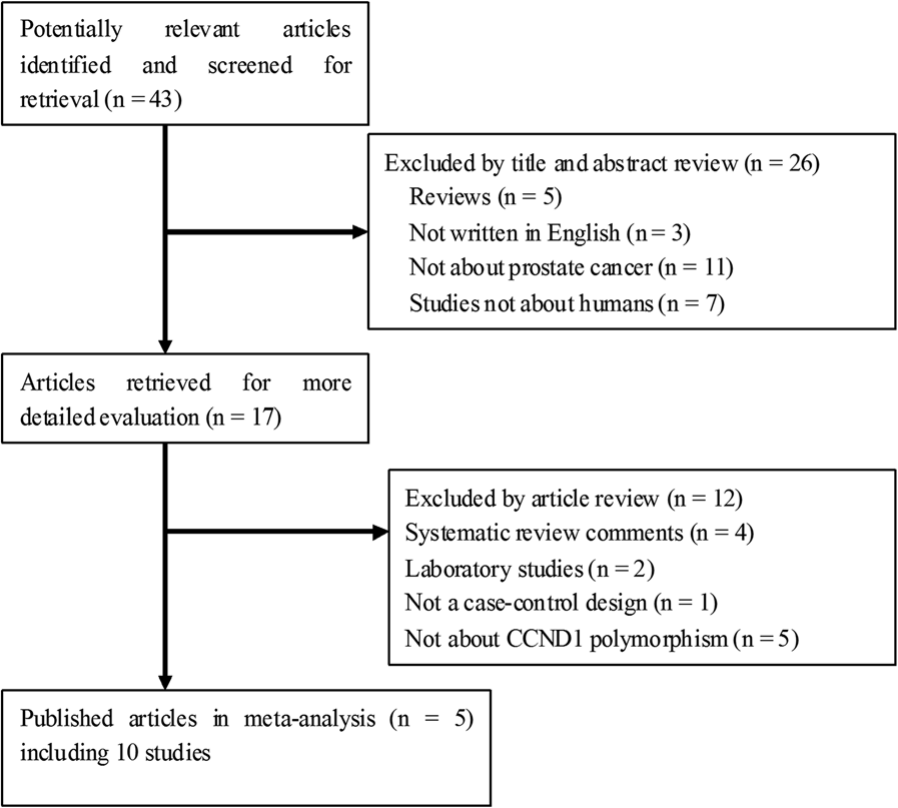
**Flow chart of study selection based on the inclusion and exclusion criteria.**

After an extensive search, a total of 43 relevant studies were identified (Figure 1). Following a careful review, five published papers with ten case-control studies were identified, with 3,820 patients with prostate cancer and 3,825 controls [10,21-24]. Table 1 presents the main characteristics of those studies, including the distribution of the various genotypes of each study in different populations. A flow diagram schematizing the process of selected and excluded articles with specific reasons for each is presented in Figure 1. All studies are case-control studies. Of these ten studies, three used polymerase chain reaction restriction fragment length polymorphism (PCR-RFLP), six used 5′-nuclease Taqman allelic discrimination assay, and one used pyrosequencing. The studies were carried out in Japan, UK, Australia, and India. Three studies were on Asians and seven studies were on Caucasians. The studies carried out with Japanese were used in the Asian subgroup, and others were used in Caucasian subgroup. The distribution of genotypes in the controls was consistent with the Hardy-Weinberg equilibrium (*P* > 0.05) in studies except for Mandal *et al.* (*P* = 0.013) and Koike *et al.* (*P* = 0.004).

**Table 1:** **CCND1 A870G genotype distribution and allele frequency in cases and controls**

**Author and year**	**Ethnicity**	**Method**	**Genotype (** ***N*** **)**						***P*** **HWE controls**
			**Case**			**Control**			
			**GG**	**GA**	**AA**	**GG**	**GA**	**AA**	
Wang *et al.* [10]	Japanese	PCR-RFLP	55	102	57	75	139	40	0.065
Mandal *et al.* [24]	Indians	PCR-RFLP	38	65	89	58	93	73	0.013
Comstock *et al.* [21]	African-Americans	Taqman	387	258	30	374	246	27	0.086
Comstock *et al.* [21]	Latinos	Taqman	212	313	118	214	315	117	0.954
Comstock *et al.* [21]	Japanese	Taqman	121	233	103	126	229	112	0.691
Comstock *et al.* [21]	Native Hawaiians	Taqman	14	40	17	12	32	24	0.814
Comstock *et al.* [21]	European Americans	Taqman	117	242	97	134	225	90	0.800
Comstock *et al.* [21]	Australians	Taqman	241	422	166	225	354	160	0.349
Kibel *et al.* [22]	Americans	Pyrosequencing	56	88	40	54	100	62	0.285
Koike *et al.* [23]	Japanese	PCR-RFLP	22	54	23	21	73	21	0.004

HWE, Hardy-Weinberg equation; PCR-RFLP, Polymerase chain reaction restriction fragment length polymorphism.

### Quantitative data synthesis

**Figure 2:**
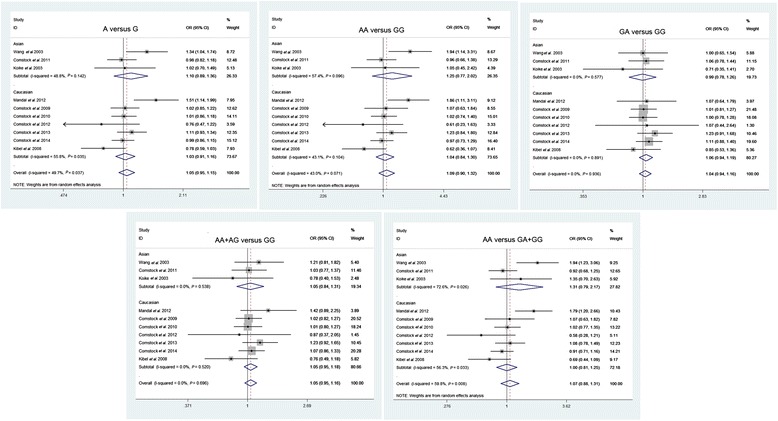
**Forest plots of the CCND1 A870G polymorphism in prostate cancer versus control and subgroup analyses.** The squares and horizontal lines correspond to the study-specific OR and 95% CI. The area of the squares reflects the weight (inverse of the variance). The diamond represents the summary OR and 95% CI. CI, confidence interval; OR, odds ratio.

Table 2 shows the results on the association between the *CCND1* A870G polymorphism and prostate cancer risk. The combined results based on all studies revealed that variant genotypes are not associated with increased prostate cancer risk in different genetic models (OR = 1.045, 95% CI = 0.947 to 1.153 for A versus G, *P* = 0.380; OR = 1.088, 95% CI = 0.896 to 1.321 for AA versus GG, *P* = 0.393; OR = 1.044, 95% CI = 0.941 to 1.158 for GA versus GG, *P* = 0.414; OR = 1.053, 95% CI = 0.955 to 1.161 for the dominant model AA + GA versus GG, *P* = 0.303; OR = 1.072, 95% CI = 0.881 to 1.306 for the recessive model AA versus AA + GA, *P* = 0.486) (Figure 2).

**Table 2:** **Meta-analysis of the association between the CCND1 A870G polymorphism and prostate cancer risk**

**Comparisons**	**Odds ratio**	**95% confidence interval**	***P*** **value**	**Heterogeneity**		**Effects model**
				***I*** ^**2**^ ** (%)**	***P*** **value**	
A versus G	1.045	0.947 to 1.153	0.380	49.7	0.037	Random
Asians	1.100	0.891 to 1.358	0.374	48.8	0.142	
Caucasians	1.026	0.911 to 1.157	0.669	55.8	0.035	
AA versus GG	1.088	0.896 to 1.321	0.393	43.0	0.071	Random
Asians	1.245	0.766 to 2.024	0.376	57.1	0.096	
Caucasians	1.043	0.835 to 1.303	0.711	43.1	0.104	
GA versus GG	1.044	0.941 to 1.158	0.414	0.0	0.936	Fixed
Asians	0.994	0.785 to 1.258	0.958	0.0	0.577	
Caucasians	1.056	0.941 to 1.186	0.350	0.0	0.891	
GA + AA versus GG	1.053	0.955 to 1.161	0.303	0.0	0.696	Fixed
Asians	1.046	0.837 to 1.308	0.690	0.0	0.538	
Caucasians	1.054	0.945 to 1.176	0.341	0.0	0.520	
AA versus GG + GA	1.072	0.881 to 1.306	0.486	59.8	0.008	Random
Asians	1.313	0.793 to 2.174	0.290	72.6	0.026	
Caucasians	1.003	0.807 to 1.248	0.976	56.3	0.033

We also performed subgroup analyses by ethnicity, and the results are listed in Table 2. The results revealed no significant associations between the *CCND1* A870G polymorphism and prostate cancer in genotype distributions in Asians and Caucasians.

In our present study, we also performed a stratified analysis of cases with PCa metastasis, and the results revealed no significant association between the *CCND1* A870G polymorphism and metastasis risk of PCa (Table 3).

**Table 3:** **Meta-analysis of the association between the CCND1 A870G polymorphism and metastasis risk of prostate cancer**

**Comparisons**	**Odds ratio**	**95% confidence interval**	***P*** **value**	**Heterogeneity**		**Effects model**
				***I*** ^**2**^ ** (%)**	***P*** **value**	
AA versus GG	1.072	0.645 to 1.782	0.788	63.3	0.066	Random
GA versus GG	0.842	0.513 to 1.382	0.496	31.2	0.234	Fixed
GA + AA versus GG	0.933	0.595 to 1.462	0.762	48.4	0.144	Fixed
AA versus GG + GA	1.230	0.824 to 1.837	0.312	53.6	0.116	Fixed

### Heterogeneity analysis

The following genetic model of the ten studies showed statistically significant heterogeneity using the *Q* statistic (A versus G: *I*^2^ = 49.7%, *P* = 0.037; AA versus GG: *I*^2^ = 43.0%, *P* = 0.071; AA versus GG + GA: *I*^2^ = 59.8%, *P* = 0.008), and the random effects model was employed in these studies. We did not find significant heterogeneity for the following model (GA versus AA: *I*^2^ = 0.0%, *P* = 0.936; AA + GA versus GG: *I*^2^ = 0.0%, *P* = 0.696), and a fixed effects model was performed.

### Publication bias

**Figure 3:**
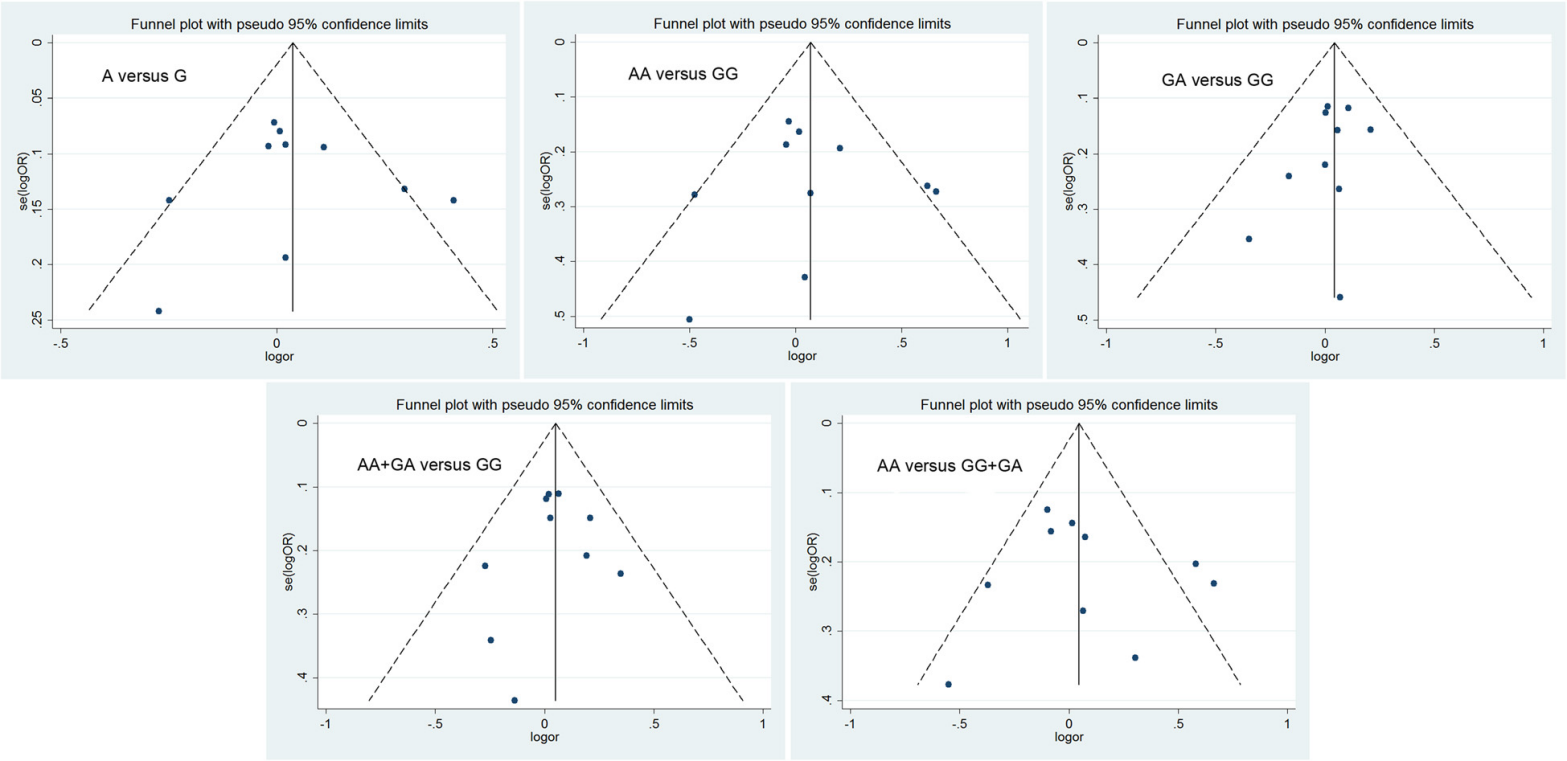
**Funnel plot was used to detect the publication bias for different genetic models.** Each point represents a separate study.

A Funnel plot and Egger’s test were performed to estimate the publication bias of the literature. The Egger-weighted regression method suggested that there was no evidence of publication bias for the *CCND1* G720A polymorphism (*P* = 0.889 for A versus G, *P* = 0.909, for AA versus GG, *P* = 0.249 for GA versus GG, *P* = 0.674 for AA + GA versus GG, *P* = 0.678 for AA versus GG + GA). This result was confirmed by the Begg’s rank correlation method (*P* = 0.721 for A versus G, *P* = 1.000 for AA versus GG, *P* = 0.371 for GA versus GG, and *P* = 0.721 for AA + GA versus GG, *P* = 0.721 for AA versus GG + GA) (Table 4, Figure 3).

**Table 4:** **Publication bias test for the CCND1 A870G polymorphism**

**Comparisons**	**Egger’s test**			**Begg’s test** ***P*** **value**
	**Coefficient**	***P*** **value**	**95% CI**	
A versus G	0.191	0.889	−3.171 to 3.554	0.721
AA versus GG	0.156	0.909	−2.899 to 3.210	1.000
GA versus GG	−0.640	0.249	−1.829 to 0.548	0.371
AA + GA versus GG	−0.329	0.674	−2.067 to 1.409	0.721
AA versus GG + GA	0.709	0.678	−3.083 to 4.500	0.721

## Discussion

Cell cycle regulation plays an important role in the evolution of cancer by influencing cell proliferation, differentiation, and apoptosis [25]. CCND1, a key regulatory protein, plays an important role in the transition from the G1 to S phase of the cell cycle [15], and its deregulation has been implicated in the pathogenesis of several types of cancers [26,27]. Many polymorphisms have been identified in *CCND1*. A common functional polymorphism, G870A (rs603965), which increased the frequency of alternative splicing and encoded a protein with an altered C-terminal domain and increased the stability or half-life of the protein, has garnered wide attention. To date, several studies have reported the role of the *CCND1* G870A polymorphism in prostate cancer risk [10,22,24]. But the results are controversial, partially because of the possible small effect of the polymorphism on PCa cancer risk. In order to estimate the overall risk of the *CCND1* G870A polymorphism associated with prostate cancer, we conducted a meta-analysis of results from ten case-control studies on the association of the *CCND1* G870A polymorphism with PCa risk. Overall, a total of 3,820 cases and 3,825 controls were included. However, the results indicated that no significant association between the *CCND1* A870G polymorphism and PCa risk was found.

Wang *et al.* [10] showed that the *CCND1 A* allele was more frequently observed in the PCa group than the control group, and men with the *AA* genotype had an increased risk of PCa compared to those with the *GG* genotype. Mandal *et al.* [24] also revealed that the *CCND1* AA genotype was observed to be associated with a significant increase in PCa risk. However, Koike *et al.* found that no significant association of the genotype frequency of the CCND1 with overall cases and controls [22]. Our present study showed that no significant association between the *CCND1* A870G polymorphism and PCa risk was found.

Stratification analysis showed that the *CCND1* A allele showed significantly increased risk of PCa metastasis [23]. But Mandal *et al.* did not find any significant risk when analyzing data for the risk of susceptibility for metastasis with the *CCND1* polymorphism [24]. In our present study, we also performed a stratified analysis of cases with PCa metastasis, and the results supported the findings that there is no significant association between *CCND1* A870G polymorphism and metastasis risk of PCa, as reported by Mandal *et al.* [24]. But the findings were inconsistent with Koike *et al.* [23]. Base on the previous studies, the reasons for the discrepancy between our study and previous studies may be various. Genetic heterogeneity is an inevitable problem in any disease identification strategy [28]. Different genetic backgrounds may cause this discrepancy, or different populations may have different linkage disequilibrium patterns. So, we hypothesized that the *CCND1* A870G polymorphism might be in close linkage with different nearby causal variants in one ethnic population but not in another, according to the report of Yu *et al.* [29]. In addition, a relatively small sample size, the genotyping method, and the prostate cancer type were also identified as potentially significant sources of between-study heterogeneity.

However, some limitations of this meta-analysis should be addressed. First, the results were based on the unadjusted estimates with original data from these collected studies being unavailable, which limited the evaluation with certain covariates, including cancer type, smoking, drinking, age, and other environmental factors. Second, the controls of several studies were various in the analysis, which may have induced the bias of the results and prevented the drawing of more detailed conclusions. Third, the single-locus-based nature of this meta-analysis precluded the possibility of gene-gene and gene-environment interactions; the analysis did not consider these factors because of the lack of sufficient data. Furthermore, we only concentrated on the *CCND1* A870G polymorphism and did not evaluate other genes or polymorphisms; whether this polymorphism integrated with other risk factors to enhance the predictive power requires further study.

## Conclusions

In conclusion, we expanded previous individually underpowered studies and suggested that no obvious association was found between the *CCND1* A870G and prostate cancer susceptibility. In addition, our observations raise the question of a potential heterogeneous effect of A870G across different ethnic populations. Nevertheless, for practical reasons, we hope that additional studies of *CCND1* that include functional DNA repair gene polymorphisms in a large cohort of different ethnicities, combined with more appropriate methods, will augment the etiology of the pathogenesis of PCa.

## Abbreviations

HWE: Hardy-Weinberg equilibrium
PCa: prostate cancer
SNP: single nucleotide polymorphism
